# Oxygen Mediates Vascular Smooth Muscle Relaxation in Hypoxia

**DOI:** 10.1371/journal.pone.0057162

**Published:** 2013-02-22

**Authors:** Jessica Dada, Andrew G. Pinder, Derek Lang, Philip E. James

**Affiliations:** Institute of Molecular and Experimental Medicine, Wales Heart Research Institute, Cardiff University School of Medicine, Heath Park, Cardiff, United Kingdom; University of Milan, Italy

## Abstract

The activation of soluble guanylate cyclase (sGC) by nitric oxide (NO) and other ligands has been extensively investigated for many years. In the present study we considered the effect of molecular oxygen (O_2_) on sGC both as a direct ligand and its affect on other ligands by measuring cyclic guanosine monophosphate (cGMP) production, as an index of activity, as well as investigating smooth muscle relaxation under hypoxic conditions. Our isolated enzyme studies confirm the function of sGC is impaired under hypoxic conditions and produces cGMP in the presence of O_2_, importantly in the absence of NO. We also show that while O_2_ could partially affect the magnitude of sGC stimulation by NO when the latter was present in excess, activation by the NO independent, haem-dependent sGC stimulator 3-(5′-hydroxymethyl-2′-furyl)-1-benzylindazole (YC-1) was unaffected. Our *in vitro* investigation of smooth muscle relaxation confirmed that O_2_ alone in the form of a buffer bolus (equilibrated at 95% O_2_/5% CO_2_) had the ability to dilate vessels under hypoxic conditions and that this was dependent upon sGC and independent of eNOS. Our studies confirm that O_2_ can be a direct and important mediator of vasodilation through an increase in cGMP production. In the wider context, these observations are key to understanding the relative roles of O_2_ versus NO-induced sGC activation.

## Introduction

Soluble guanylate cyclase (sGC) is well known as the “receptor” for nitric oxide (NO). Binding of this gaseous diatomic molecule to the haem moiety of the enzyme stimulates the conversion of guanosine triphosphate (GTP) to cyclic guanosine monophosphate (cGMP), a nucleotide that is involved in several vital intracellular signalling cascades and physiological processes [1]. Although NO is the preferred ligand for sGC, activating the enzyme several hundred fold over its basal level, other gaseous and synthetic activators, such as carbon monoxide (CO) and the benzylindazole derivative 3-(5′-hydroxymethyl-2′-furyl)-1-benzylindazole (YC-1) respectively, have been identified. While only a moderate activator, CO would seem to mediate its action via the same mechanism as NO. YC-1 on the other hand employs a totally different strategy, binding to an allosteric site on the enzyme thereby increasing the maximal catalytic rate. Such an action produces a 10-fold increase in basal sGC activity independently of other ligands, while also potentiating the responses to subsequent exposure to such agents [2]. Given the above it is evident that there are more ways than one to activate sGC. Importantly the physiological relevance of actions that modulate the response of sGC to other ligands remains in question.

Perhaps one of the most significant roles for the NO/sGC axis is in the control of vascular tone [3]–[5], a function that is essential to maintaining blood flow and oxygen/nutrient delivery to tissues. As such, in response to NO produced by adjacent endothelial cells, sGC located within vascular smooth muscle is activated and the subsequent production of cGMP mediates vasorelaxation. Since CO can also induce this cGMP-dependent response, it is perhaps a little surprising, given that NO, CO, and molecular oxygen (O_2_) differ by only one valence electron, that a role for O_2_ in this process has not been identified.

Conventional understanding would suggest that O_2_ simply does not bind to sGC [6]–[8], at least not in the same way as NO and CO. However changes in O_2_ tension are widely recognised to influence vascular tone. For instance, via the regulation of specific potassium channel activity, O_2_ plays a major role in the control of pulmonary vascular tone [9], [10]. Perhaps of more relevance to an interaction with sGC is our previous demonstration that the relaxation response to endogenous NO stimulation or exogenous NO addition is enhanced the lower the O_2_ tension [11], implying an inverse relation between NO effectiveness and O_2_. These factors contribute to the concept of “hypoxic vasodilation”, an innate physiological response designed to maintain tissue perfusion in the face of falling O_2_ concentrations [12]–[16]. While the exact mechanisms that underlie this response have been the subject of active research and debate for many years, it is now widely accepted that activation of sGC is intrinsically involved.

The introduction of oxygenated red blood cells (RBCs) to hypoxic tissues is now well recognised to immediately induce vasorelaxation. However, while these cells would certainly, and very quickly, release O_2_ under such conditions, the latter has been overlooked as a potential mediator of the relaxation response in favour of more conventional activators of s’GC. To this end, oxygenated haemoglobin-derived nitrosothiol (HbSNO) [17], nitrite (NO_2_^−^)-derived NO as a consequence of deoxyhaemoglobin nitrite reductase activity [15], [20], and RBC-derived adenosine triphosphate (ATP) stimulation of endothelium-dependent release of NO and prostacyclin [21], [22] have all been postulated to mediate “hypoxic vasodilation”.

While all or any of the above could contribute to RBC-induced vasodilation in the acute setting, it would seem that none provides a totally clear mechanism. The data described in this manuscript advocates an alternative and more straight-forward candidate, molecular O_2_. Therefore, the aim of the present study was to investigate whether O_2_ could act as a possible direct ligand for sGC and/or a modulator of the actions of other preferred ligands. To accomplish these aims two model systems were used; an isolated sGC enzyme system and an isolated blood vessel system, both of which allowed for tight control of the local O_2_ environment. Importantly, these models not only allowed us to probe O_2_-dependent mechanisms but also to investigate how these align with the known features of the putative sGC-dependent relaxants described above.

## Materials and Methods

### Ethics Statement

#### Animal

In accordance with the United Kingdom Animal (Scientific Procedures) Act of 1986, this study did not require a Home Office project license because no regulated procedures were carried out. White, male New Zealand rabbits were humanely killed at a designated establishment by sodium pentobarbitone, which is an appropriate method under Schedule 1 of the Act.

#### Human

Methods requiring human blood samples were fully approved by the local research ethics committee (South East Wales Research Ethics Committee) and the joint NHS/University Research and Development Office (Cardiff and Vale University Health Board). All healthy volunteers gave written informed consent.

### Materials

L-N^G^-monomethyl Arginine (L-NMMA) and 1H-[1], [2], [4] oxadiazolo-[4, 3-a] quinoxalin-1-one (ODQ) were from Alexis Biochemicals. Soluble guanylate cyclase (sGC) purified enzyme was from Axxora as ≥90% pure α_1_β_1_. Methylamine hexamethylene methylamine (MAHMA) NONOate (NOC-9) was from Enzo Life Sciences. 2, 3-diphosphoglycerate (2, 3-DPG), YC-1, PEG-SOD (polyethylene glycol superoxide dismutase) and PEG-CAT (catalase) were from Sigma Aldrich.

### Isolated sGC Study

All experiments were performed in an Invivo_2_ Hypoxia Workstation 400 (Ruskin). Normoxic experiments were carried out at 37°C and 20% O_2_/5% CO_2_ via a 25% O_2_/5% CO_2_ gas cylinder (BOC). Hypoxic experiments were maintained at 37°C and ∼0% O_2_/5% CO_2_. Reagents were allowed to equilibrate for 1 hour before all tests were completed. Precise buffer O_2_ concentrations for these experiments are also shown in Table 1.

**Table 1:** Example O_2_ contents of replica buffer samples for both the myograph and hypoxic chamber, read from a PDT-probe standard curve (EPR oximetry).

Sample	Stock Buffer O_2_ concentration (µmol/L)	Final Tissue Bath concentration(µmol/L)^1^
0%	49	1.96
21%	175	7.0
95%	772	30.9
Hypoxic chamber 0% +1 hour	38	
Hypoxic chamber 20% +1 hour	197	

1 represents the effective O_2_ concentration added to each tissue bath. The existing O_2_ in the “hypoxic” bath perfused with N_2_/CO_2_ was 0.9% (equivalent to 9 µmol/L O_2_).

The pure sGC enzyme was reconstituted in buffer (Tris 50 mmol/L, DTT 1 mmol/L, 0.5% BSA, pH 7.4) and diluted 1 in 200 in assay buffer (Tris 50 mmol/L, EGTA 100 µmol/L, MgCl_2_ 0.3 mmol/L, 0.045% BSA, pH 7.4. GTP, dissolved in equimolar MgCl_2_, was then added to a final concentration of 1 mmol/L to start the reaction. As appropriate, NOC-9 (to give final concentrations of 0.118, 1.118, 11.8 and 118 µmol/L) or YC-1 (100 µmol/L) were added at the same time as the GTP. In experiments utilising superoxide dismutase (SOD) and catalase (CAT), agents were added 60 minutes prior to GTP. Hyperoxic samples were prepared by perfusing the buffer-enzyme mix with 95% O_2_/5% CO_2_. All reactions were incubated for 10 minutes at 37°C, after which boiling inactivation buffer (Tris 50 mmol/L, EDTA 4 mmol/L, pH 7.5) was added in 4 times excess. Samples were then heated to boiling point before storage at −20°C for further analysis.

### cGMP ELISA

Sample cGMP content was measured by commercial ELISA (R&D Systems) as directed in the kit manual.

### Isolated Vessel Preparation

Male New Zealand White rabbits (2–2.5 kg) were killed by a lethal dose of sodium pentobarbitone (120 mg/kg, i.v) under Schedule 1 guidelines (see ‘Ethics statement’). Subsequently the thoracic aorta was carefully removed and placed in fresh Krebs buffer (composition (mmol/L): NaCl 109.2, KCl 2.7, KH_2_PO_4_ 1.2, MgSO_4_ 1.2, NaHCO_3_ 25, Glucose 11, CaCl_2_ 1.5) on ice. Excess adipose tissue was removed from the aorta and 8 rings, 2 mm in width, were prepared.

For isometric tension recording, rings were suspended in tissue baths containing 5 ml of Krebs at 37°C and gassed with 95% O_2_/5% CO_2_. Resting tension was set to 2 g. Signals from the transducer (AD Instruments, Chalgrove, UK) were amplified and visualised on the Powerlab/Chart 4 for Windows software. All rings were allowed to equilibrate for 60 minutes prior to experimentation.

Constriction-relaxation exercises to 1 µmol/L phenylephrine (PE) and 10 µmol/L acetylcholine (ACh) (Sigma Aldrich), respectively, were performed in order to establish both smooth muscle and endothelial integrity.

In order to equilibrate the rings in defined levels of hypoxia, the gas supply was switched to the appropriate O_2_ content (ensuring balance of N_2_ and 5% CO_2_) for 10 minutes. Constriction to PE was tested across a broad range of concentrations and the appropriate PE concentration to achieve 80% of maximal attainable constriction used in all studies. For hypoxic studies (0% O_2_), rings were typically exposed to 3 µmol/L PE in order to achieve a similar sub-maximal constriction as observed in normoxic rings. The O_2_ concentration measured in the tissue bath equilibrated under hypoxic conditions was 0.9% (equivalent to 9 µmol/L O_2_) as we have detailed previously [11].

### Krebs Samples

Krebs solution was gassed at 95% O_2_/5% CO_2_ in order to achieve 95% O_2_ samples. 21% O_2_ samples were allowed to equilibrate with the atmosphere. 0% O_2_ samples were gassed with 95% N_2_/5% CO_2_. In order to accurately measure O_2_ concentration within these equilibrated Krebs buffer samples, electron paramagnetic resonance (EPR) oximetry was undertaken utilising N^15^ per-deuterated tempone (2,2,6,6-tetra-methyl-4-piperidone; PDT) as the O_2_-sensitive probe. In brief, the spectral line width obtained from PDT (∼5 µmol/L) shows a linear relationship with O_2_ concentration and sample readings can be measured against known standards. This technique measures O_2_ with an accuracy of ±0.2% O_2_ in our hands as we have described in detail previously [23]. The precise O_2_ concentrations within “95%”, “21%” and “0%” buffer are shown in Table 1.

### Haemoglobin (Hb) Samples

Venous RBCs were lysed and diluted 1 in 3 in PBS (0.9% sodium chloride w/v), of which 250 µl was then loaded on to a Sephadex G25 M column (GE Healthcare). Samples of pure Hb were collected in fractions for use in the myograph studies. The HbO_2_ content was verified by blood gas analysis as described (see ‘Venous RBC samples’). The volume used in the myograph studies gave a final O_2_ concentration in the tissue bath of 30.9 µmol/L which was equal to the O_2_ content of the tissue bath following addition of either buffer or RBC samples.

### Venous RBC Samples

Blood was drawn from the antecubital vein of healthy volunteers and immediately centrifuged at 1200×g for 5 minutes. The plasma layer and buffy coat were removed and replaced with an equivalent volume of gassed (95% O_2_/5% CO_2_) phosphate buffered saline (PBS). Following re-centrifugation at 1200×g for 5 minutes the PBS layer was removed. In order to achieve RBCs of varying O_2_ saturation, the cells were firstly diluted ∼1∶3 with PBS and loaded on to a thin film rotating tonometer, which was purged with O_2_/CO_2_ to achieve higher saturations or N_2_/CO_2_ for lower saturations as we have described previously [24]. The O_2_ saturation of representative RBC samples was measured on an OSM3 Hemoximeter (Radiometer, Copenhagen).

### *In vitro* NO Analysis

Phosphate buffered saline (PBS), pH 7.4, was kept at 37°C in a reaction vessel purged with a flow of nitrogen gas feeding into a Nitric Oxide Analyser (NOA) 280i (Analytix) for on-line ozone-based chemiluminescence (OBC) detection of NO. NOC-9 was reconstituted in 0.1M sodium hydroxide (NaOH) to give a final stock concentration of 24 mmol/L and kept on ice in the dark. For the experimental samples, the NOC-9 was further diluted in 1 ml of PBS (protected from light in a sealed vessel) to give the following concentrations (in µmol/L): 2.4; 1.2; 0.24; 0.12 and 0.024. In order to compare NO release under different conditions, the PBS was either pre-equilibrated to 0% or 95% O_2_ by vigorous bubbling. Samples were incubated for 10 minutes at 37°C, after which 200 µl of the gas layer was drawn up using a Hamilton syringe and injected immediately into the purge vessel for OBC analysis. After the signal trace returned to baseline values, 200 µl of the corresponding PBS sample was drawn up and injected into the purge vessel. Data were recorded in real time and presented as area under the curve (Liquid software).

### Statistical Analysis

All data were analysed by one-way ANOVA plus Tukey’s or Dunnett’s *post hoc* test or Student’s *t* test as appropriate. Pearson’s correlations were assessed and coefficients are shown where appropriate. All data are expressed as mean ± standard error (SEM). Differences were considered significant where p<0.05.

## Results

### Isolated Pure sGC Activity in Normoxia/Hypoxia

**Figure 1:**
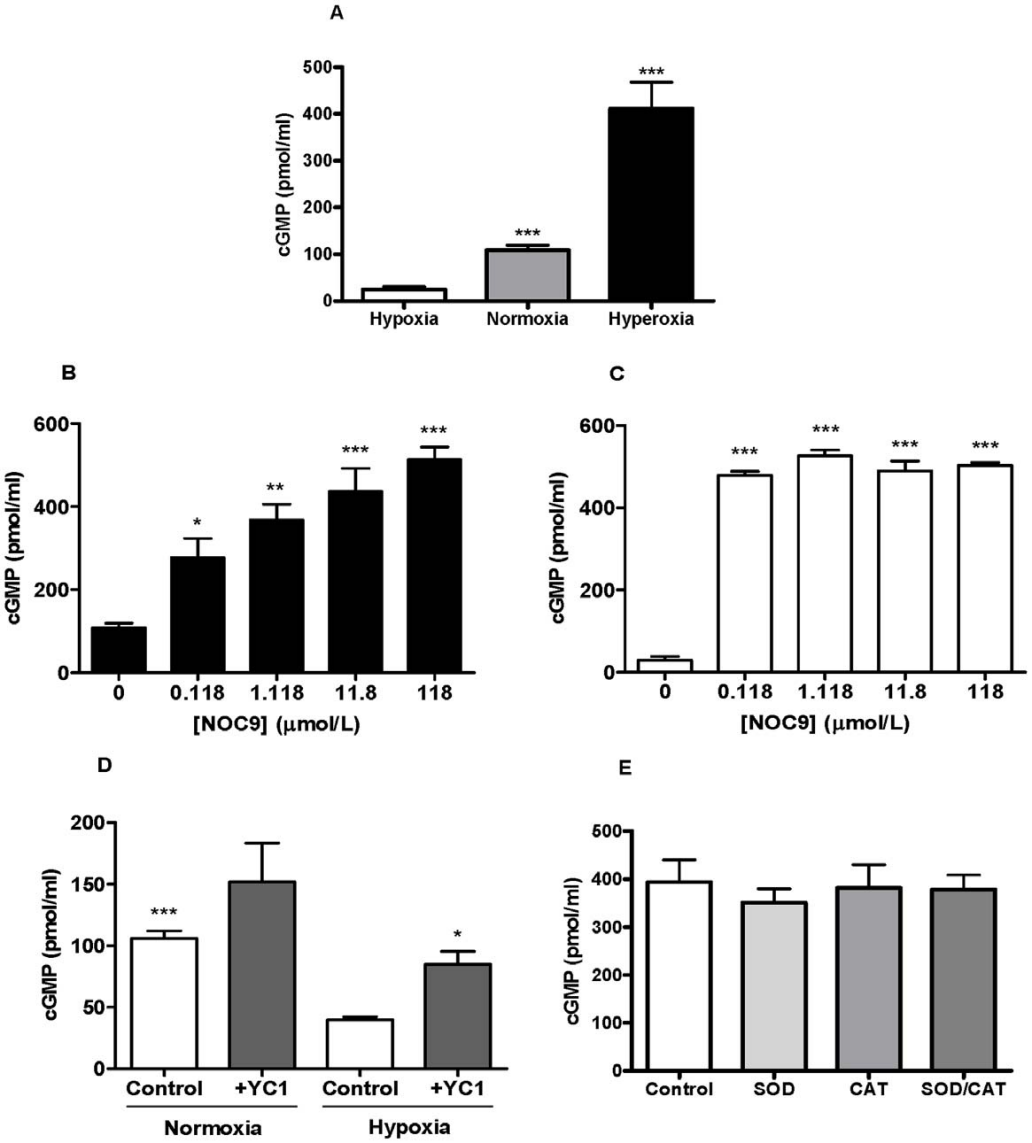
Measurement of cGMP as an index of sGC activity in normoxic and hypoxic conditions. a. Comparing cGMP production in hypoxia (0% O_2_), normoxia (∼21% O_2_) and hyperoxia (95% O_2_). Normoxia and hyperoxia stimulated an increased production of cGMP compared with hypoxia. (***p<0.001 cf. hypoxia, n = 4–6). b. Concentration response curve to NOC9 under normoxic conditions. Significant differences were observed at each concentration level compared to the control containing no NOC9, ***p<0.001 cf. 0 µmol/L, **p<0.01 cf. 0 µmol/L and *p<0.05 cf. 0 µmol/L (n = 3 to 6 experiments). c. Concentration response curve to NOC9 under hypoxic conditions. Significant differences were observed at each concentration level compared to the control containing no NOC9, ***p<0.001 cf. 0 µmol/L (n = 3 to 6 experiments). cGMP detected for 0.118 and 1.118 µmol/L NOC9 were much greater than the levels in normoxia initiated by the same concentrations. d. Exposure of sGC to the NO-independent activator YC-1 (100 µmol/L). Paired analysis of cGMP levels catalysed by sGC revealed a significant increase in the normoxic control versus hypoxic control, ***p<0.001 cf. hypoxia control. The addition of YC-1 in normoxia induced an increase in cGMP compared to control but this was not significant. A similar trend was observed in hypoxia, however the addition of YC-1 induced a significant rise in cGMP, *p<0.05 cf. control. (n = 4). e. SOD (100 U/ml) and CAT (250 U/ml) incubated with sGC in normoxia stimulated a similar level of cGMP compared to the control, p>0.05 cf. control. (n = 4).

A striking observation of these experiments was the difference in basal sGC activity in normoxia (∼100 pmol/ml) compared to hypoxia (∼30–40 pmol/ml) (Figure 1a).

Exposure to the NO donor, NOC-9, at various concentrations (0, 0.118, 1.118, 11.8 and 118 µmol/L), stimulated cGMP production in normoxia in a concentration dependent manner (108.4±10.78 pmol/ml to 513.7±30.12 pmol/ml) (Figure 1b). In hypoxia, cGMP production was similar across all concentrations of NOC9 compared with the control (∼500 pmol/ml vs. ∼30 pmol/ml) (Figure 1c).

In separate experiments, YC-1 (100 µmol/L), a compound known to stimulate sGC without direct interaction with the sGC haem group [2], was added to sGC under hypoxic and normoxic conditions as above. While the addition of YC-1 enhanced the amount of cGMP produced by the enzyme, importantly the increase from baseline was similar in both normoxia and hypoxia (∼46.20 pmol/ml & ∼44.86 pmol/ml, respectively) (Figure 1d).

To deduce whether superoxide (O_2_^−^) was having a direct influence on sGC activity in normoxia, SOD and CAT were incubated with the enzyme to observe any change in cGMP levels produced. Compared to control levels, SOD and CAT did not significantly affect the activity of the isolated enzyme (Figure 1e).

### Effects of Buffer Containing Increasing O_2_ on Vascular Tone in Hypoxia

**Figure 2:**
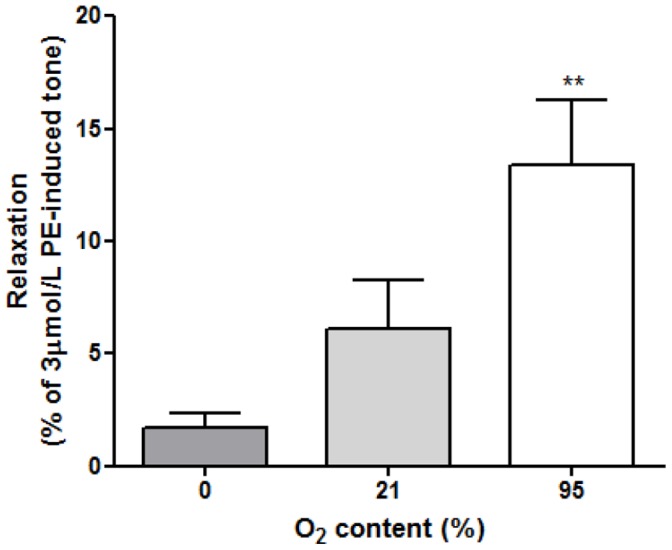
Krebs buffer samples introduced to hypoxic aortic rings. Maximum relaxation was demonstrated with 95% oxygenated buffer samples compared with 0% O_2_ samples (all n = 6, **p<0.01 cf. 0%).

Tetrameric Hb has the ability to carry four O_2_ molecules [25]. The O_2_ content of a RBC suspension is therefore much greater than buffer equilibrated with the same O_2_ concentration. In order to attain a comparable O_2_ delivery, Krebs buffer was gassed with 95% O_2_/5% CO_2_, either in a sealed glass bottle (high O_2_ saturation) or an open container allowed to equilibrate with atmospheric air (21% O_2_), or with 95% N_2_/5% CO_2_ (0% O_2_). 200 µl of the appropriate Krebs buffer samples were then added to tissue baths containing hypoxic PE-pre-constricted aortic rings as described above. A 3 fold greater (p<0.01) relaxation was observed following addition of highly oxygenated versus minimally oxygenated samples (Figure 2).

### Effects of Administering a Constant O_2_ Supply to Hypoxic Tissue

In separate experiments, aortic tissue was incubated in hypoxia and pre-constricted with PE as described above. The gas supply was then switched to 95% O_2_/5% CO_2_ permanently. An instantaneous and transient relaxation response, akin to that following addition of O_2_ buffer solution, was observed (data not shown).

### Effects of sGC Inhibition on O_2_-mediated Vasorelaxation

**Figure 3:**
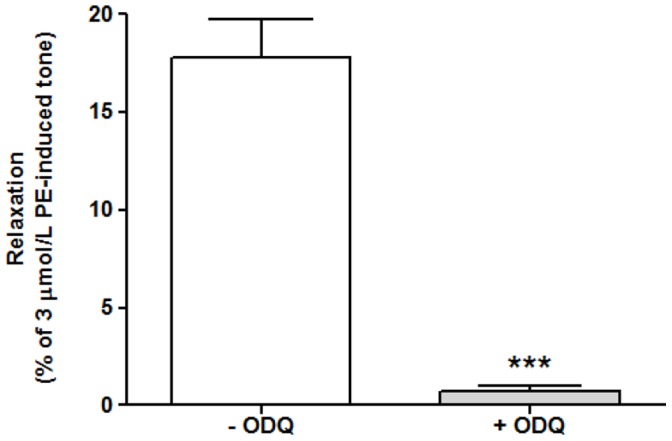
The effect of sGC inhibitor, ODQ, upon O_2_-mediated relaxation. In the presence of ODQ (10 µmol/L) the relaxation response to 95% oxygenated buffer was almost completely inhibited (both n = 7, ***p<0.001 cf. control).

To confirm that O_2_-mediated relaxation of hypoxic vessels is mediated via the sGC pathway, the sGC inhibitor, 1H-[1], [2], [4] oxadiazolo-[4, 3-a] quinoxalin-1-one (ODQ) which binds to the haem moiety of sGC, was introduced 30 minutes prior to PE constriction. The presence of ODQ caused a significant inhibition of the relaxation induced by a bolus of 95% O_2_/5% CO_2_-gassed Krebs buffer (0.72±0.28% vs. 17.81±1.90% in the absence of ODQ, n = 7, ***p = 0.001) (Figure 3).

### Influence of Endothelial NO Synthase (eNOS) on O_2_-mediated Vasorelaxation

**Figure 4:**
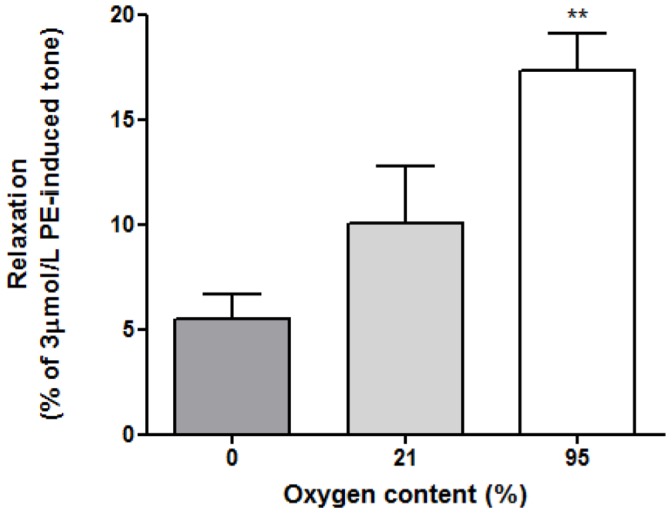
The influence of eNOS inhibitor, LNMMA, upon O_2_-mediated relaxation. LNMMA had no effect on the relaxation responses. Similarly to Figure 3, 95% oxygenated buffer samples generated the greatest relaxation compared with 0% O_2_ samples (all n = 4, **p<0.01 cf. 0% O_2_).

Experiments were repeated in the presence of the eNOS inhibitor L-N^G^-monomethyl arginine (LNMMA, 300 µmol/L for 30 minutes). Figure 4 demonstrates that this agent has no effect on the oxygenated buffer-induced relaxations (n = 4, **p<0.01 cf. 0% O_2_).

### Vascular Effects of Superoxide and Hydrogen Peroxide

**Figure 5:**
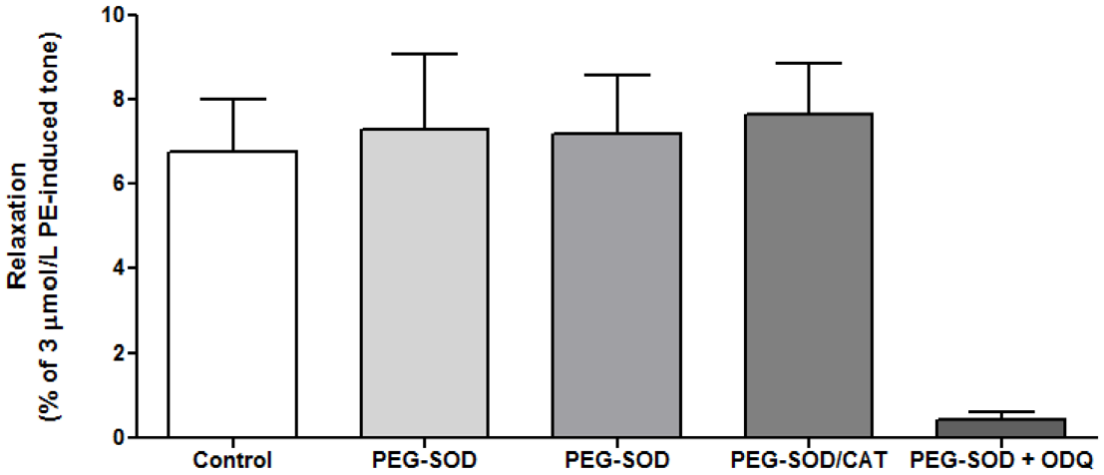
Effect of O_2_^−^ and H_2_O_2_ on vessel relaxation in hypoxia. The inhibition of O_2_^−^ radicals (100 U/ml PEG-SOD) and H_2_O_2_ (250 U/ml PEG-CAT) within the vessel system did not affect the magnitude of the relaxation to a bolus of 95% oxygenated buffer. PEG-SOD (100 U/ml) plus ODQ (10 µmol/L) abolished most of the relaxation (*p<0.05 cf. control). All n = 3.

It is well acknowledged that reperfusion of O_2_ to hypoxic vessels causes the generation of O_2_ radical species [26]. Therefore, it was necessary to ascertain whether O_2_^−^ had a role in the myography experiments conducted here. SOD and CAT linked to polyethylene glycol (PEG) were therefore used to abrogate any effects of intracellular O_2_^−^ and hydrogen peroxide (H_2_O_2_) generation, respectively. Neither PEG-SOD, PEG-CAT, nor both together had a significant effect on the O_2_-induced relaxation of vessels in hypoxic conditions (Figure 5).

### Effects of Oxygenated RBCs on Vascular Tone in Hypoxia

**Figure 6:**
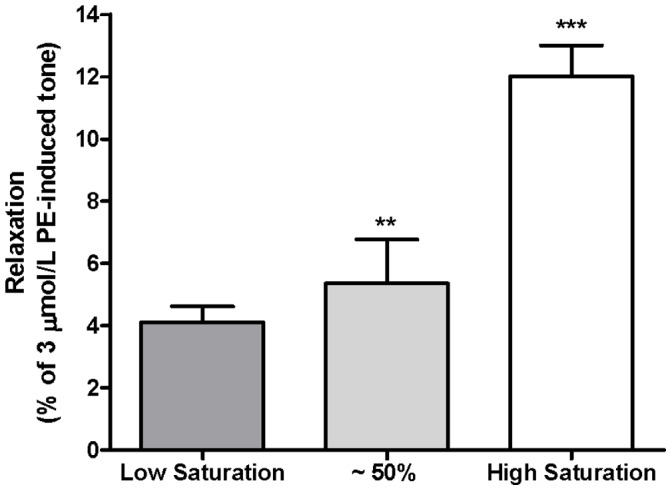
Varying O_2_ saturations of RBC administered to hypoxic rabbit aortic rings. Partially saturated RBC (∼50%) and low saturated RBC displayed a significantly lower relaxation than RBC of high saturation, **p<0.01 and ***p<0.001 respectively. (High saturation, n = 13; partial saturation, n = 4; low saturation, n = 13).

RBCs equilibrated at a higher O_2_ saturation (98.22±0.45% O_2_, n = 13) caused significantly (p<0.001) more relaxation than those of partial saturation (51.43±6.16% O_2_, n = 4) or low saturation (20.40±5.28% O_2_, n = 13) when administered to a hypoxic tissue bath (Figure 6). These data confirm a positive relationship between RBC O_2_ saturation and the relaxation induced in hypoxic aortic tissue (Pearson’s correlation coefficient r = 0.815, p<0.0001).

### Effects of RBC O_2_ Cycling upon Hypoxic Vasorelaxation

Highly oxygenated RBC (98.22±0.45% O_2_, n = 13) were administered to hypoxic aortic rings to induce relaxation. In a parallel sample, highly oxygenated RBC were first deoxygenated to ∼20% O_2_ (20.68±1.66% O_2_) before reoxygenation (97.70±0.39% O_2_, n = 4) and subsequent addition to hypoxic rings. There was no significant difference in the relaxation induced post deoxy-oxy cycling of RBCs compared with controls (8.28±2.97% vs. 12.00±3.60%).

The RBC O_2_ cycling prompted further investigation into the movement of the relaxing moiety in and out of the cell. RBC were deoxygenated to a low saturation (22.38±1.69% O_2_) before re-suspension in fresh oxygenated buffer to give a saturation of ∼98% (98.05±0.65% O_2_, n = 4). As above, there was no significant difference between the relaxations induced by buffer-replaced RBCs compared with controls (12.97±1.89% vs. 12.00±3.60%).

### Influence of 2, 3-diphoshoglycerate on RBC-mediated Hypoxic Vasorelaxation

2, 3-diphosphoglycerate (2, 3-DPG), (5 mmol/L) discourages O_2_ from binding to Hb, facilitating diffusion into respiring tissue [27]. In the presence of 2, 3-DPG, hypoxic PE-pre-constricted aortic rings relaxed significantly more (10.75±1.11% vs. 8.25±1.36%, n = 4, *p<0.05) to addition of oxygenated RBCs than in its absence, supporting the role of this intermediate in the regulation of O_2_ delivery.

### Comparison of O_2_-mediated Vasorelaxation by RBCs, Buffer or Isolated Hb

**Figure 7:**
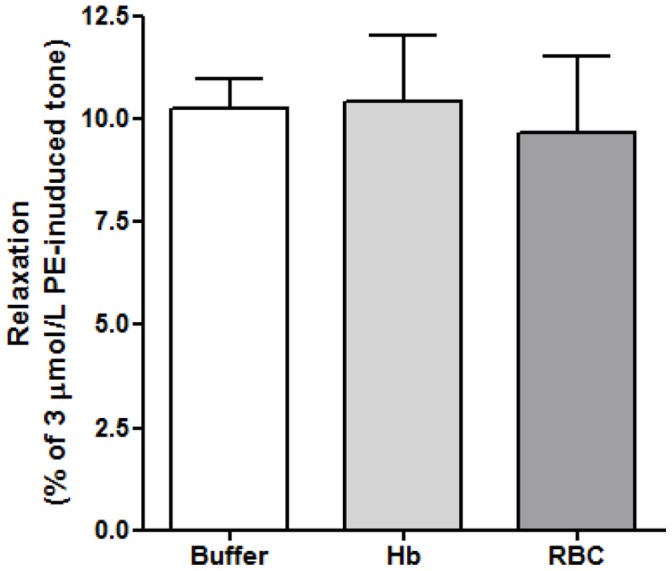
Effects of various oxygenated samples on relaxation responses of hypoxic vascular tissue. Krebs buffer, Hb and RBC samples of equal O_2_ content (∼30 µmol/L) administered to hypoxic rabbit aortic rings. There was no difference in the magnitude of the relaxations produced (p>0.05) (n = 4).

In additional experiments, purified oxygenated Hb was prepared from RBCs and applied to tissue baths containing hypoxic PE-pre-constricted aortic rings as described above. Samples of RBC, buffer or Hb were prepared such that addition to the tissue bath resulted in final O_2_ concentrations of ∼30 µmol/L. Figure 7 shows grouped results confirming very similar relaxation responses for RBCs, Hb and oxygenated buffer (n = 4, p>0.05).

### Effects of Oxygenated RBCs on Vascular Tone at Different Tissue pO_2_

**Figure 8:**
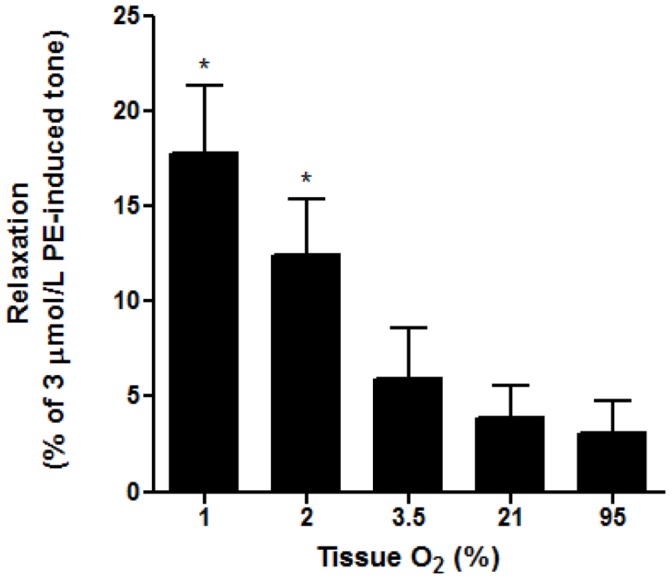
Relaxations observed after addition of RBC samples to aortic rings equilibrated at various O_2_ saturations. Tissues held at a lower O_2_ (1 and 2%) relaxed significantly more to oxygenated RBC’s, ***p<0.001 cf. 95% and *p<0.05 cf. 95%, respectively.

In separate experiments, aortic tissue was perfused for 10 minutes with 1%, 2%, 5%, 21% or 95% O_2_/5%CO_2_. Constriction to PE was normalised to 80% of maximum at each O_2_ tension, then fully oxygenated RBCs were administered. This is in contrast to most of the experiment described above in which varying O_2_ was introduced to hypoxic tissue or isolated sGC. Significantly greater relaxation was induced at tissue incubated in 1% (***p<0.001 cf. 95% O_2_) and 2% O_2_ (*p<0.05 cf. 95% O_2_) (n = 6) (Figure 8).

### Effects of O_2_ on the Liberation of NO from NOC9

**Figure 9:**
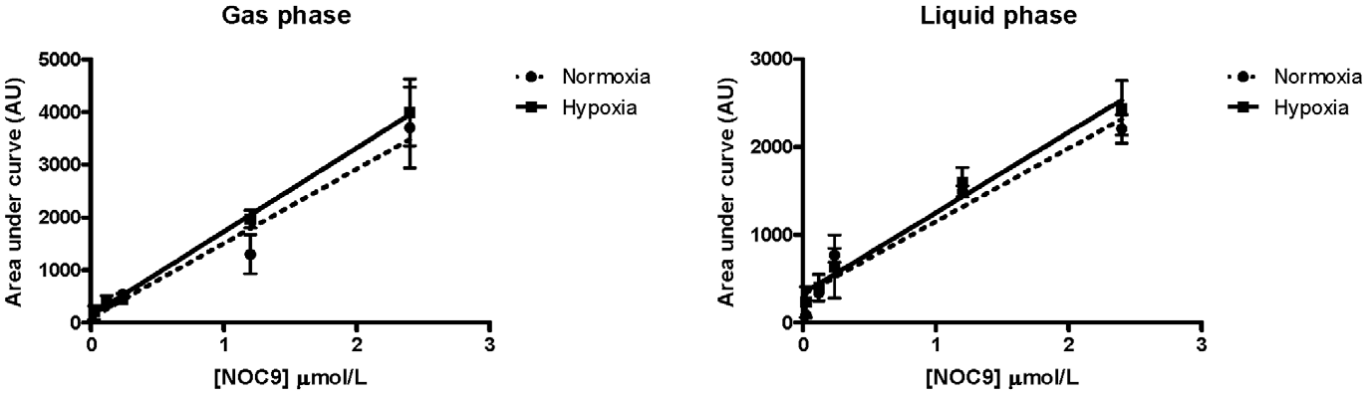
NO liberation from NOC-9 under normoxic versus hypoxic conditions. Gas and liquid samples drawn up from reaction vessels incubated with varying concentrations of NOC-9, were injected into the NOA. The solutions were either equilibrated at 0 or 95% O_2_/5% CO_2_ prior to the addition of NOC-9. There was no difference in the total NO detected in either gas or liquid samples under (n = 3, p<0.05).

In order to test whether production of NO from NOC-9 was affected under normoxic or hypoxic conditions, we measured NO released from NOC-9 into both the gaseous and liquid phases of a PBS sample across a range of NOC-9 concentrations. Our data confirms that the quantity of NO detected in both the gas and liquid phases (quantified as area under the curve), are not significantly different under normoxic versus hypoxic conditions (n = 3, p<0.05) (Figure 9).

## Discussion

The aim of the present study was to investigate the effects of O_2_ on sGC function, and in particular, on responses of the enzyme to known ligands and stimulators/activators. Our data conclusively shows that molecular O_2_ has the ability to increase sGC activity in the absence of NO. Moreover, exposure of sGC to hypoxic conditions revealed a novel modulatory effect of O_2_ on NO-stimulated sGC activity. Our isolated vessel model confirms that the sGC-mediated transient relaxation of tissues in hypoxic conditions can occur simply by injecting a bolus of oxygenated buffer. As such, the data presents an important and hitherto undescribed role for O_2_ in the production of cGMP.

As previously described, and when compared to NO, sGC can be activated to a lesser extent by other diatomic species such as CO [28]. However, despite the obvious structural similarity, the ability of O_2_ to interact with sGC, and specifically with the haem moiety to form a stable complex, has been overlooked [6]–[8], [29], [30]. Importantly, the data described herein goes against conventional thinking and supports a role for O_2_ in upregulating baseline sGC activity as evidenced by greater cGMP production in our isolated enzyme system under normoxic compared to hypoxic conditions. Moreover, the presence of O_2_ is also shown to have a modulatory effect on the subsequent activation of sGC by NO. Thus following addition of NO to hypoxic sGC, similar large increases in cGMP were observed irrespective of NO concentration. Conversely, a concentration dependent response to NO was revealed for normoxic sGC, the largest response to 23.6 µmol/L NO being similar to that produced by 100-fold less NO under hypoxic conditions. As such, the removal of O_2_ from sGC both decreases baseline enzyme activity and makes it more sensitive to activation by lower concentrations of the NO. That the NO and haem-independent sGC activator, YC-1, produced a different response, similar increases in cGMP above baseline being observed in both normoxia and hypoxia, would support a possible role for the interaction of O_2_ with the sGC in a way that modulates the haem group. sGC has been reported to function optimally at intracellular O_2_ concentrations between 20 and 40 µmol/L [31] indicating the ability of sGC to distinguish between ligands. Importantly, we confirmed that the amount of NO released by the NO donor NOC-9 was similar under both hypoxic/normoxic experimental conditions and thus cannot be accountable for the results achieved. While a stable isolatable complex of O_2_ and sGC has not been identified, it is possible that intracellular concentrations of O_2_ could have an influence on enzyme activity. The majority of studies conducted with sGC are under anaerobic conditions [8], [32], [33] however Ullrich and colleagues [34] observed a decrease in cGMP produced by platelet sGC following N_2_ addition compared to basal conditions. In addition, Soret band analysis by UV spectrophotometry show subtle changes in sGC binding under oxygenated conditions [35].

In order to substantiate our isolated enzyme data, we next developed an *in vitro* vascular model. Isolated rabbit aortic rings pre-constricted with PE under hypoxic conditions were exposed to single bolus additions of buffer equilibrated with varying O_2_ concentrations. In such experiments, transient and concentration-dependent relaxation responses to O_2_ were produced. The involvement of sGC and classic cGMP-dependent downstream signalling in smooth muscle to induce relaxation was confirmed by the fact that the O_2_-dependent responses were inhibited in the presence of ODQ, the irreversible haem-site inhibitor of sGC [36], [37]. Since eNOS function is close to normal even under hypoxic conditions in this vascular preparation [38] a role for endothelium-dependent NO in the responses described above was also investigated. In that the relaxation induced by O_2_ was insensitive to eNOS inhibition by LNMMA, such an involvement was ruled out. Together these observations support the role of O_2_ in sGC activation already described above.

The *in vitro* isolated vascular system used in the present study relates closely to ischaemia reperfusion, where a vessel of low oxygenation quickly becomes reperfused with O_2_, for example following removal of an occlusion [39]. Under such circumstances many studies have shown evidence for the generation of free radical species [26], [40]–[43]. Taken with the recently observed inhibitory effect of O_2_^−^ on sGC [44] it was important to confirm that O_2_^−/^H_2_O_2_ were not involved in the sGC-driven effects we observe following addition of O_2_ to the tissues. To this end experiments were repeated in the presence of the appropriate cell permeable inhibitors (PEG-SOD and PEG-CAT). That the relaxation responses to O_2_ were again insensitive to these agents ruled out this possibility.

In order to further translate our study, we repeated our *in vitro* studies, substituting the O_2_ buffer bolus for highly O_2_-saturated human RBCs. When oxygenated, RBCs have the ability to change Hb conformation into the R state. Indeed, in this state Hb has the capacity to offload O_2_ and *in vivo* this is conveyed by the transfer of O_2_ from the blood to respiring tissue beds. Importantly, such a mechanism is crucial for RBCs to give up O_2_ when encountering a hypoxic environment. Therefore, by altering the saturation of RBCs, we could change the proportions of R and T state Hb and therefore potentially show the preference for the RBCs to give up O_2_ in the R state. The highly saturated (98.22±0.45% O_2_) RBCs induced a significantly greater relaxation of hypoxic rabbit aortic rings compared with RBCs of low saturation (20.40±5.28% O_2_)/mainly T state Hb. Consequently, purified Hb was used in further experiments to confirm the relaxant species was released directly from Hb without the need for RBCs *per se*. Interestingly, RBCs, Hb and buffer boluses of comparable O_2_ content induced very similar relaxations of the hypoxic vessel tested in the present study. Additional experimentation demonstrated that the relaxant species is not depleted by repeat deoxygenation/re-oxygenation cycles, confirming the species is readily replenishable and is essentially delivered by simply replacing the Krebs buffer surrounding the hypoxic RBCs with adequately oxygenated buffer.

For some time it has been suggested that RBC-induced vasorelaxation is linked to Hb allostery, although a particular interest has focussed on the now well established vasodilatory capacity of HbSNO. We ourselves followed this line of investigation demonstrating greater RBC-induced relaxations with increasing HbSNO [45], and an altered HbSNO to relaxation response in highly glycosylated RBCs that we linked to an effect on the Hb O_2_ saturation curve [11]. Concurrently, we also measured the transient release of O_2_ into the tissue bath but at the time took this to be a consequence of Hb transition rather than the relaxant species. Subsequent studies have later confirmed the potential for O_2_ in buffer alone to induce relaxation, but considered this control considerably less than the relaxation induced by RBCs [15]. Importantly, the samples and the buffer controls were perfused with the same gas, but crucially were not equalised in terms of O_2_ content in the latter study.

Further studies have confirmed the importance of Hb allostery in RBC-mediated hypoxic vasorelaxation [20], [46] although they contend partially oxygenated Hb acts as a NO_2_^−^ reductase that converts readily available NO_2_^−^ to free NO to exhibit vasodilatation. Whilst both HbSNO and NO_2_^−^ exhibit properties that suggest they could contribute to RBC-induced relaxation [15], [18]–[20], [45], neither completely satisfies the characteristics of a readily replenishable, freely diffusing relaxant species that is present in simply oxygenated buffer as we show here.

A potential criticism of our model system is that we induce an artificial condition in tissue whereby a defined unit of O_2_ (either buffer or RBCs) is introduced to an essentially hypoxic environment. To address this, we conducted studies under conditions where either fully oxygenated RBCs or buffer samples were administered to baths containing tissue equilibrated at various O_2_ concentrations. Of physiological significance, O_2_-mediated relaxation was inversely related to the extent of tissue oxygenation, in complete concurrence with established HbO_2_ delivery and matching the need for dilation across the whole O_2_ range. Furthermore, we conducted studies in which O_2_ was confirmed as a relaxant of blood vessel segments since the first response to administration of a constant supply of O_2_ being re-introduced into a hypoxic setting was transient relaxation.

In previous work we had demonstrated how vessel relaxation response to exogenous addition of NO is enhanced with lower O_2_ tension [11]. This had been ascribed to the influence of O_2_ on competing mechanisms, including direct effects on constriction. However, taken together with the data we show herein, would indicate a more direct interplay between the effects of NO and O_2_ on sGC. Clearly, NO is the preferred ligand as shown by its ability to fully activate isolated sGC in our model system at a concentration as low as 10 µmol/L even in the presence of 210 µmol/L O_2_ (“normoxia”). Under conditions where NO is relatively low, O_2_ may influence sGC in two ways; the influence of weak sGC activation by O_2_ may accrue greater importance, and the effect of NO will be enhanced.

In conclusion, our data has revealed the novel finding that in the presence of O_2_, sGC can stimulate an increased production of cGMP from GTP compared to under hypoxic conditions. The use of YC-1 in our isolated enzyme model clearly enhanced cGMP production however the compound was not affected by the presence of O_2_, perhaps suggesting the effects of O_2_ on the enzyme may be occurring at a site independent to that of YC-1. Furthermore, we show that O_2_ can modulate the effect of NO (concentration-dependent) upon the enzyme, indicative of an interaction of O_2_ with the haem of sGC or an indirect involvement with haem via an allosteric site. Armed with the knowledge that ∼6% of sGC within vascular tissue is stimulated by NO under normal conditions [47], our findings may have important consequences in the wider context of vascular relaxation via stimulation of sGC across the physiological and pathological O_2_ range.
